# Combination therapy of ursodeoxycholic acid and glucocorticoid and (or) immunosuppressant in patients with primary biliary cholangitis: A meta-analysis

**DOI:** 10.1097/MD.0000000000028987

**Published:** 2022-03-04

**Authors:** Zi-Long Wang, Kai-Min Song, Rui Jin, Yan-Di Xie, Yu-Qiong Wang, Zhi-Cheng Liu, Bo Feng

**Affiliations:** a Peking University Hepatology Institute, Peking University People's Hospital, Beijing, China; b The Fifth Clinical Medical College of Shanxi Medical University, Jinzhong, Shanxi, China; c Peking University China-Japan Friendship School of Clinical Medicine, Beijing, China.

**Keywords:** glucocorticoid, immunosuppressant, meta-analysis, primary biliary cholangitis, ursodeoxycholic acid

## Abstract

**Background/objectives:**

Primary biliary cholangitis (PBC) is a chronic inflammatory autoimmune cholestasis liver disease. There were many studies comparing a combination of glucocorticoids and/or immunosuppressants to a single UDCA therapy in PBC patients, while the literature demonstrated divergent finds. To evaluate the effectiveness of ursodeoxycholic acid (UDCA) combined with glucocorticoids and (or) immunosuppressants on biochemistry, immunology, histology, clinical symptoms, and adverse reactions of PBC from the perspective of evidence-based medicine.

**Materials and methods:**

PubMed, web of science, the Cochrane Library, EMBASE databases were searched to collect clinical randomized trials and self-control studies of UDCA combined with glucocorticoids and (or) immunosuppressants and UDCA monotherapy in the treatment of PBC. The retrieval time is from the establishment of the database to August 2020. Two reviewers independently screened literature, extracted data and evaluated the bias of included studies. Revman 5.3 software was used for meta-analysis.

**Results:**

Six studies including 201 patients were included. The meta-analysis found that the combination therapy can improve some biochemical indexes, immunological indexes, and clinical symptoms of patients with PBC. However, combination therapy has no significant improvement in other biochemical indicators which respond to liver and bile duct damage, such as ALT, GGT, and ALB. Besides, the improvement of liver histology is limited, and the incidence of adverse events is higher.

**Conclusion:**

Overall, the combination therapy showed no improvement in key biochemical parameters and limited improvement in liver pathology. Besides, the side effects were more serious. Therefore, in the current treatment regimen, it is not recommended for PBC patients.

## 1 Introduction

Primary biliary cholangitis (PBC) is a chronic inflammatory autoimmune cholestasis liver disease, and its pathogenesis may be related to the interactions of genetics, immunity, and bile pathways.^[1]^ Data from multiple research centers showed that one in 1000 women over 40 years old worldwide had PBC.^[2]^ It was previously believed that PBC was relatively rare in China. However, with the deepening understanding of this disease and improvement of testing technology, the prevalence of PBC has been reported to be rising rapidly in recent years,^[3]^ which we should put more energy into clinical treatment. Currently, the first-line drug for PBC is ursodeoxycholic acid (UDCA) which requires long-term or even lifelong medication. About 40% of the patients have a poor response to UDCA,^[4]^ therefore, additional drugs are needed to provide added benefits.

Glucocorticoids and immunosuppressants are the most commonly prescribed agent for inflammatory and autoimmune diseases. In recent years, many studies have suggested that UDCA combined with immunosuppressive drugs are beneficial, but there are still studies that hold different views.^[5,6]^ Differences in research results may come from the small number of patients included or the different population of patients included, in which some were initially treated and others were poor responses. Therefore, it is still controversial whether UDCA combined with glucocorticoids and (or) immunosuppressants can improve the prognosis of patients with PBC. Therefore, we conducted a meta-analysis, with suitable inclusions and exclusions, to evaluate the efficacy of therapies combining UDCA and Glucocorticoids and (or) immunosuppressants compared with those of a UDCA monotherapy for PBC patients.

## 2 Methods

### 2.1 Study identification

This study was based on the Preferred Reporting Items for Systematic Reviews and Meta-Analysis (PRISMA) statement.^[7]^ Computer retrieval of PubMed, Web of Science, the Cochrane Library, EMBASE databases, dating from the establishment of the database to August 2020, and the search content is the clinical research literature of UDCA and Glucocorticoids and (or) immunosuppressants. The English words: “primary biliary cholangitis,” “primary biliary cirrhosis,” “glucocorticoids,” “immunosuppressants,” “ursodeoxycholic acid,” and “randomized controlled trial”; At the same time, the dissertation, conference summary, and clinical trials from Clinical Trials.gov were also retrieved. Ethical approval and patient conset were not required, as this study was done on published data.

### 2.2 Inclusion criteria

The studies included in this meta-analysis fit the 4 criteria.

1. Study population: all patients were diagnosed as PBC, which could be diagnosed if 2 of the following 3 criteria were met:^[8]^
  1. Biochemical evidence of cholestasis;
  2. Other specific antibodies including Sp100 or gp210 were positive when antimitochondrial antibody was positive or negative;
  3. Histological evidence showed nonsuppurative destructive cholangitis and destruction of the interlobular bile duct.
2. Study design: the control group was treated with UDCA monotherapy, while the experimental group was treated with immunosuppressants and/or glucocorticoids based on UDCA therapy. Except for the different treatment options, the characteristics of patients were similar and comparable.
3. Research data: the data is complete and reliable, which can accurately extract the mean and standard deviation (Mean ± SD).
4. Research Type: clinical randomized controlled trials (RCT); self-controlled clinical trial.

### 2.3 Exclusion criteria

1. Controlled trials of immunosuppressive agent monotherapy;
2. Additional drugs based on combination therapy of UDCA and immunosuppressive agents and/or glucocorticoids;
3. Retrospective literature and repeated published literature;
4. The research data is incomplete or expressed in image representation.

### 2.4 Data extraction

The data were independently searched, read, and screened according to the inclusion criteria by the 2 researchers (Zi-Long Wang and Kai-Min Song), and further discussion was conducted by consensus if there was any dispute. The researchers independently extracted the literature with a unified data extraction table, cross-checked the collected information, and consulted a third party for adjudication if there was any disagreement. If the data is incomplete, try to contact the author for information. If the data is expressed in terms of quartile spacing, then we transform it through a mathematical formula for subsequent meta-analysis.^[9]^ The data extraction table included literature sources, research methods, research object information, research intervention, and other information.

### 2.5 Methodological quality

The Jadad scale was used to evaluate the randomized controlled trials and self-controlled trials, which included 3 major parts: randomization, double-blind, exit, and loss of follow-up. This is a five-point quality scale, the score less than or equal to 2 is defined as low quality, and the score more than 3 is defined as high quality. The quality evaluation was completed by 2 researchers independently.

### 2.6 Statistical analyses

All statistical analyses were performed with RevMan 5.3 software. *X* ^2^ test and *I* ^2^ test were used to detect heterogeneity, and a *P* value of <.10 or *I* ^2^ value >50% was considered as substantial heterogeneity. A fixed-effects model was used when the heterogeneity test showed a *P* value of >.10 and an *I* ^2^ value <50%; otherwise, a random-effects model was used. When the heterogeneity is too large, sources of heterogeneity were explored through sensitivity analysis and subgroup analysis. Besides, funnel plots were constructed to evaluate the presence of publication bias.

## 3 Results

### 3.1 Descriptive and qualitative assessments

**Figure 1:**
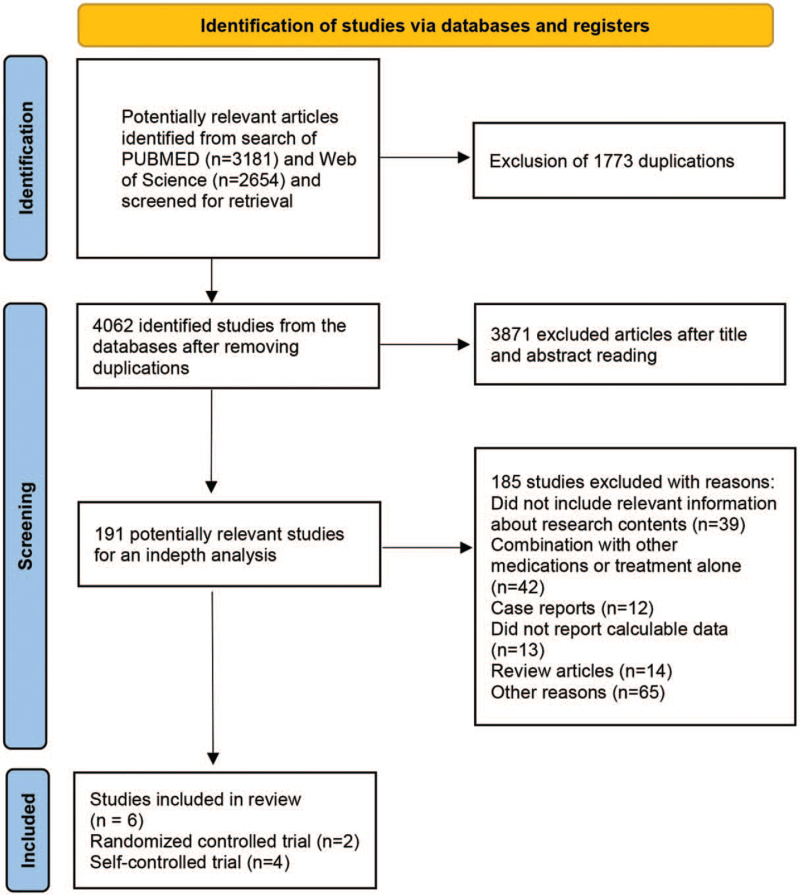
the flowchart of the included study.

After excluding reviews, case reports, and other unrelated literature, we finally selected 6 studies from 4062 studies (Fig. 1). Not all studies were published as full-text articles, 1 trial was published as an abstract. These studies involved 195 patients, the mean age of patients ranged from 42.3 to 52.3 years and the mean duration of treatment ranged from 6 to 36 months. The daily dose of UDCA ranged from 10 to 15 mg/kg, and the drugs for combination therapy were budesonide or methotrexate, or mycophenolate mofetil. The baseline characteristic of 6 trials were shown in Table 1. The quality of the included literature was evaluated by Jadad scores. Unfortunately, 4 of the studies were self-controlled trials, leading to low scores in the evaluation of method quality.

**Table 1:** Baseline characteristics of the trials included in the meta-analysis.

					Duration of treatment		
References	Monotherapy (n)	Combination Therapy (n)	UDCA Dose (mg/d)	Combination Drug	UDCA	COM	Publication type
Angulo et al^[6]^	22	22	13–15	Budesonide	At least 6 m	12 m	Full text
Rautiainen et al^[10]^	37	37	15	Budesonide	36 m	36 m	Full text
Buscher et al^[13]^	8	8	10–15	Methotrexate	At least 6 m	6 m	Full text
Rabahi et al^[14]^	15	15	13–15	Budesonide, MMF	At least 12 m	36 m	Full text
Angulo et al^[11]^	25	19	13–15	MMF	66.4 m	12 m	Abstract
Rautiainen et al^[12]^	32	30	15	Budesonide	36 m	36 m	Full text

### 3.2 Meta-analysis

#### 3.2.1 ALP levels

**Figure 2:**
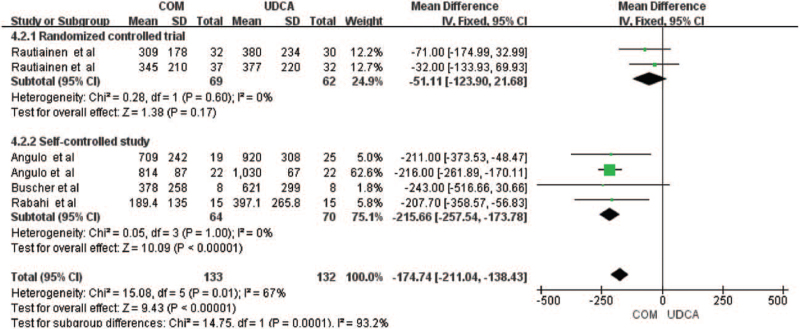
Alkaline phosphatase levels according to the type of study of patients treated with monotherapy versus combination therapy for PBC. COM = combination therapy, CI = confidence interval, df = degree of freedom, IV = inverse-variance, UDCA = ursodeoxycholic acid.

Six trials including 201 patients reported data regarding the endpoints of ALP levels,^[6,10–14]^ showing that combination could significantly reduce the ALP levels of PBC patients (mean difference [MD] = −174.74, 95% confidence interval [CI] = −211.04, −138.43, *P *< .00001), with significant heterogeneity between the 2 groups (*I* ^2^ = 67%, *P* = .01). According to the type of article, the subgroup was divided into the RCT and self-controlled trial subgroup analysis. It was found that the combined treatment of the RCT subgroup could not reduce the ALP level of PBC patients (MD = −51.11, 95% CI = −123.90, 21.68, *P* = .17, Fig. 2). The internal heterogeneity of each subgroup was 0, and the difference between subgroups was statistically heterogeneous (*I* ^2^ = 93.2%, *P* = .0001).

#### 3.2.2 TBIL

**Figure 3:**
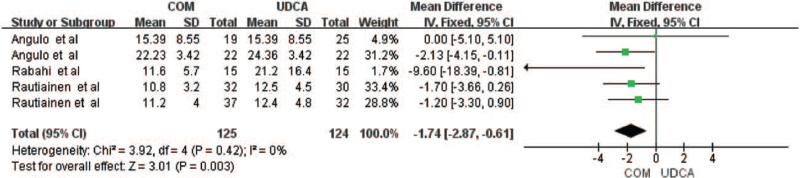
Total bilirubin levels of patients treated with monotherapy versus combination therapy for PBC. CI = confidence interval, df = degree of freedom, IV = inverse-variance.

Five trials including 193 patients reported data regarding the endpoints of TBIL levels,^[6,10,11,13,14]^ showing that combination could significantly reduce the TBIL levels of PBC patients (MD = −1.74, 95% CI = −2.87, −0.61, *P* = .003, Fig. 3), with no significant heterogeneity between the 2 groups (*I* ^2^ = 0%, *P* = .42).

#### 3.2.3 Clinical symptoms

**Figure 4:**
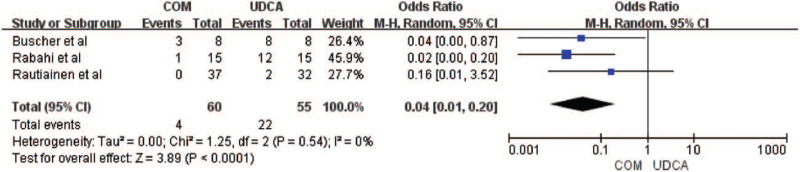
Effects of monotherapy versus combination therapy on clinical symptoms in patients with PBC. CI = confidence interval, df = degree of freedom, IV, inverse-variance, M-H = Mantel-Haenszel.

Three trials including 92 patients reported symptoms of pruritus, fatigue, and other symptoms before and after treatment,^[10,13,14]^ showing that the combination therapy improved the clinical symptoms of the patients (MD = 0.04, 95% CI = 0.01, 0.20, *P* < .0001, Fig. 4), there was no significant heterogeneity between the 2 groups (*I* ^2^ = 0%, *P* = .54).

#### 3.2.4 Adverse events

**Figure 5:**
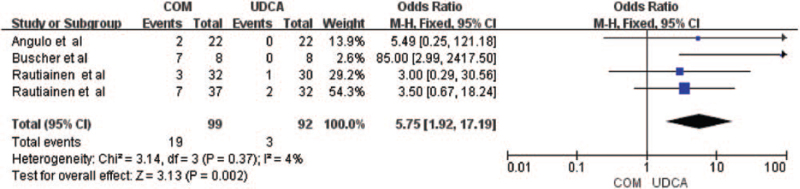
Adverse events in PBC patients treated with monotherapy versus combination therapy. CI = confidence interval, df = degree of freedom, M-H = Mantel-Haenszel, IV = inverse-variance.

Four Trials including 161 patients reported data on the adverse events,^[6,10,12,13]^ showing that combination therapy increased adverse events in PBC patients (OR = 5.75, 95% CI = 1.92, 17.19, *P* = .002, Fig. 5), and there was slight heterogeneity between the 2 groups (*I* ^2^ = 4%, *P* = .37).

### 3.3 Publication bias

**Figure 6:**
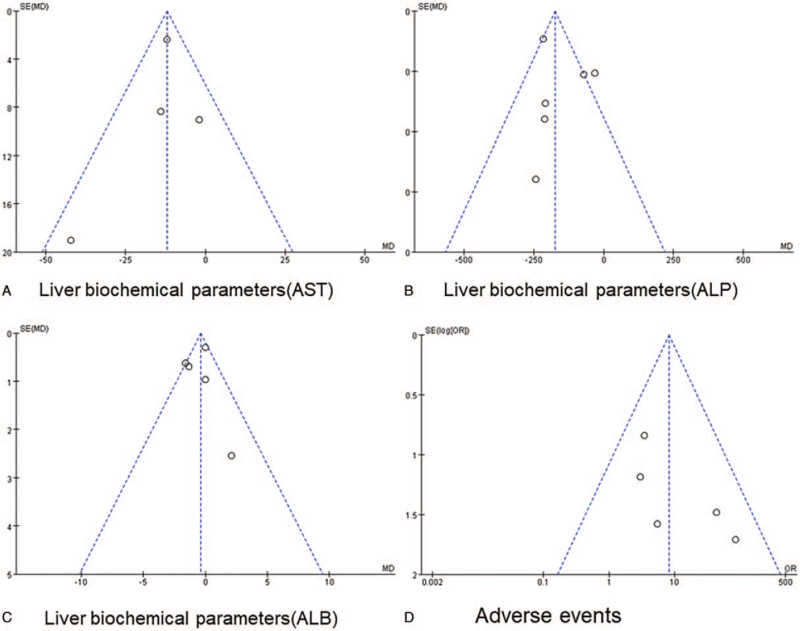
Funnel plots for the meta-analysis.

The indicators of 4 or more articles included in the study were analyzed by funnel plot (Fig. 6). The funnel plots of the meta-analysis for liver biochemical parameters, clinical symptoms, and adverse events showed slight asymmetry, suggesting possible publication bias (Supplemental doc).

## 4 Discussion

Although UDCA is the-first line drug for the treatment of PBC patients proven by many trials, 40% of patients still have a poor response to it.^[4]^ As an autoimmune disease, there are a lot of immunosuppressants and glucocorticoids being investigated in PBC, which has never stopped for nearly 30 years. The pathogenesis of PBC is closely related to immune disorder. The increased CD8^+^ T cells, monocytes, and NK cells in the liver suggest that innate immunity and adaptive immunity play an important role in PBC.^[15]^ While immunosuppressants act on the patients by regulating the number and function of lymphocytes. Glucocorticoid is also effective on cholestatic liver disease. One of the mechanisms of glucocorticoids’ effect may be to reduce the expression of a bile acid synthesis rate-controlling enzyme, Cyp7a1, to inhibit the synthesis of bile acid in the liver; besides, it can also increase the expression of hepatocyte basolateral bile transporter (NTCP) and apical sodium-dependent bile acid transporter (ASBT) to promote the absorption and uptake of bile acid.^[16]^ However, there was a contradiction among the results from different countries and scholars, which made doctors confused about whether immunosuppressive agents should be used. It is necessary to summarize and analyze the previous articles, so we conducted this meta-analysis.

There are few studies on PBC patients who used immunosuppressive therapy, glucocorticoids and immunosuppressants play the role of immunosuppression, thus the 2 types of drugs were both to be included in this article. In addition, there are few RCT studies about the immunosuppressants in the treatment of PBC, and some studies constructed tables and graphs to present the data, which made it difficult to extract accurate data. Therefore, RCT was included together with the self-controlled trial. Due to differences in the design for the different types of trails, the level of evidence varies, and there is heterogeneity in methodology. For the outcome indicators with high heterogeneity, sources of heterogeneity were explored through subgroup meta-analyses, and the heterogeneity can be improved after subset analysis. In addition, RCT-type articles included patients with initial treatment of PBC, while other literature included PBC patients with poor UDCA response, which may also be the source of heterogeneity.

Compared with UDCA monotherapy, the AST, ALP, TBIL, IgG, and IgM levels were significantly attenuated by combination therapy in PBC patients, but there were no significant changes of serum biochemical index such as ALT and GGT. Due to the large heterogeneity of ALP indicators, subgroup analysis was conducted for different research types, which showed that the combination therapy did not significantly improve the ALP level in RCT. It was easy to understand why there was not any significant change in ALP levels. The number of literatures included was small, and the patients were initially treated which showed that the combination therapy had not significantly improved the ALP level, but evidently improved the patients with poor UDCA response. For patients with poor UDCA response, the baseline ALP index was high at the time of inclusion, and some patients may have autoimmune hepatitis (AIH) characteristics at the same time, this may lead to being sensitive to immunosuppressive therapy. However, due to the lack of access to the original data for analysis, further verification is still needed. A recently published RCT about budesonide combined with UDCA in the treatment of PBC in patients with poor UDCA response also showed that the combination therapy can significantly reduce the ALP level of patients, suggesting that the combination therapy has a better improvement effect on ALP and TBIL than a single drug in patients with poor UDCA response.^[17]^ Due to the long publication time of the literature, models for judging prognosis were not mentioned, such as Paris I standard,^[18]^ Paris II standard,^[19]^ GLOBE score,^[20]^ and UK-PBC score.^[21]^ Therefore, we mainly judge the prognosis by biochemical indicators and liver histology. ALP and TBIL are widely accepted as prognostic factors of PBC. The GLOBAL PBC research group found that the recovery of TBIL and ALP after UDCA treatment was significantly correlated with the risk of liver transplantation and death.^[22]^

There is a view that immunosuppressive agents are effective in improving liver histology.^[12,14]^ Conversely, some studies show that the improvement of liver histology is limited,^[5,17,23]^ the difference between the 2 viewpoints may result from different standards. Analysis was conducted on the histological judgments, showing that combination therapy made little improvement in liver histology by using mostly Ludwig histological staging,^[24]^ while other studies show that combination therapy could improve the histology may use more detailed criteria. That is one reason why the results were different, more detailed criteria meant histological changes were observed more easily. Another possible cause might be that compared with the patients with liver pathology at baseline, the number of patients with liver pathology at the end of the study significantly reduced, resulting in the deviation of the result. Therefore, large sample, long-term RCT studies are still needed to verify the benefit of combination therapy on liver pathology.

Adverse drug reactions were described in different literature, including aggravation of fatigue, osteoporosis, gastrointestinal discomfort, Cushing's syndrome, pulmonary toxicity, oral ulcer, alopecia, and so on. However, it is certain that the side reactions in combination therapy on PBC patients were significantly higher than UDCA monotherapy, suggesting that combination therapy has certain risks. Budesonide has been highly anticipated for its high first-pass effect and low incidence of adverse events. However, in a recent three-year randomized, placebo-controlled trial, it was found that budesonide could not improve the histology of PBC patients with poor UDCA response, and nearly two-thirds of the patients had serious adverse reactions,^[17]^ suggesting that the risk of serious adverse events is still unbearable compared with the benefits of biochemical index improvement. In the current treatment methods of PBC, the benefits of obeticholic acid and fibrate drugs are significantly higher than those of immunosuppressive agents, so it is not recommended to use them clinically.

Our study still presented some limitations. First, limited data were available on the efficacy of combination therapy in PBC, and some data cannot be extracted.^[25]^ Second, there is a natural defect that the quality level of self-controlled trials is lower than that of RCT, so the Jadad scale scores were too low. Besides, there needs a medicine washout period between pre and post medication, but as the first-line medication of PBC is UDCA, it is not recommended for patients to quit the UDCA treatment, so there is no drug washout period in the middle. A previous study of 203 PBC patients found that a biochemically stable state could be achieved after UDCA treatment for half a year and remained stable during subsequent follow-up.^[26]^ For the question of whether the efficacy of combination therapy may be the delay of monotherapy effect, the studies of UDCA treatment for at least half a year were included. Finally, some of the research data were represented by interquartile distance, which was inconsistent with the input form of Meta-analysis, so the calculation formula is adopted for conversion.^[9]^ Although this method has been evaluated and can be applied to evidence-based medicine, it still has defects compared with the original data.

## 5 Conclusion

Through the data of PBC patients from 2 RCTs and 4 self-control trials, a comprehensive and quantitative analysis was conducted on whether PBC patients could be treated with glucocorticoids or immunosuppressants. First, compared with UDCA monotherapy, combination therapy has no significant improvement in some biochemical indicators which respond to liver and bile duct damage, such as ALT, GGT, and ALB. Second, for the clinical symptoms of PBC patients, the combination therapy has a significant improvement compared with UDCA monotherapy. Third, the results of different literature on the improvement of liver histology are different, suggesting that the improvement of liver histology needed further validation. Last, considering the obvious adverse reactions of glucocorticoids and immunosuppressants, it is suggested that the combination of glucocorticoids and immunosuppressants is not an effective and viable treatment for PBC patients. These data provide an important reference value for the future research of immunosuppressive therapy on PBC patients.

## Author contributions

**Data curation:** Zilong Wang.

**Formal analysis:** Bo Feng, Rui Jin.

**Investigation:** Yandi Xie.

**Methodology:** Kanmin Song, Yuqiong Wang, Zhicheng Liu.

## Supplementary Material

Supplemental Digital Content
