# Supplementary material for: Combination therapy of ursodeoxycholic acid and glucocorticoid and (or) immunosuppressant in patients with primary biliary cholangitis: A meta-analysis
Source: Medicine (Baltimore). 2022 Mar 4;101(9):e28987. doi: 10.1097/MD.0000000000028987 (PMC8896518; doi:10.1097/MD.0000000000028987)
Supplement: Supplemental Digital Content [file medi-101-e28987-s001.doc]

**ALT and AST levels**

Three trials including 92 patients reported data regarding the endpoints of ALT levels 9-11, showing that combination therapy did not reduce ALT levels in patients with PBC (MD=-11.36 IU/L, 95%CI: -32.06 9.34, P =0.28, Figure S1). The results showed a high degree of heterogeneity (I2 = 70%, P = 0.03). By analyzing the source of heterogeneity, it was found that the index of the control group in the study [7] was higher than that in other literature, which was the source of heterogeneity.

Four trials including 70 patients reported data regarding the endpoints of AST levels6, 10-12, showing that combination therapy significantly reduced AST levels in PBC patients (MD=-11.94, 95%CI =-16.31, -7.58, P <0.00001, Figure S1), with no significant heterogeneity between the two groups (I2=21%, P =0.28).

Figure S1: Alanine transaminase and aspartate transaminase levels of patients treated with monotherapy versus combination therapy for PBC.


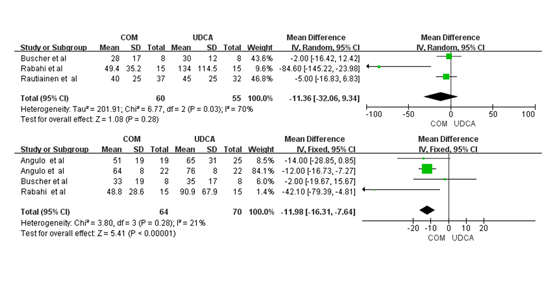
Abbreviations: UDCA, ursodeoxycholic acid; COM, combination therapy; SD, standard deviation; IV, inverse-variance; CI, confidence interval; df, degree of freedom; PBC, primary biliary cholangitis; ALT, Alanine transaminase; AST, Aspartate transaminase.

**GGT levels**

Two trials including 77 patients reported data regarding the endpoints of GGT levels6, 9, showing that combination did not reduce the level of GGT in PBC patients (MD=-33.48, 95%CI =-111.84, 44.88, P =0.40, Figure S2), with no significant heterogeneity between the two groups (I2=0%, P =0.93).

Figure S2: γ-Glutamyl Transferase levels of patients treated with monotherapy versus combination therapy for PBC.


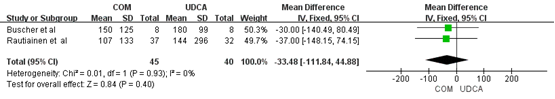


Abbreviations: UDCA, ursodeoxycholic acid; COM, combination therapy; SD, standard deviation; IV, inverse-variance; CI, confidence interval; df, degree of freedom; PBC, primary biliary cholangitis; GGT, γ-Glutamyl Transferase.

**ALB**

Six trials including 201 patients reported data regarding the endpoints of ALB levels6, 10-13, showing that combination did not significantly reduce the ALB levels of PBC patients（MD=-0.37，95% CI=-0.85，0.10, P=0.12）, with moderate statistical heterogeneity between the two groups ( I2=40%, P=0.14). According to the type of article, the subgroup was divided into RCT and self-controlled trial subgroup analysis. It was found that the combined treatment of the RCT subgroup could have a lower ALB level of PBC patients (MD =-1.47, 95%CI =-2.38, -0.55, P =0.002, Figure S3). The internal heterogeneity of each subgroup was 0, and the difference between subgroups was statistically heterogeneous (I2=86.7%, P = 0.006).

Figure S3: Albumin levels of patients according to the type of study treated with monotherapy versus combination therapy for PBC.


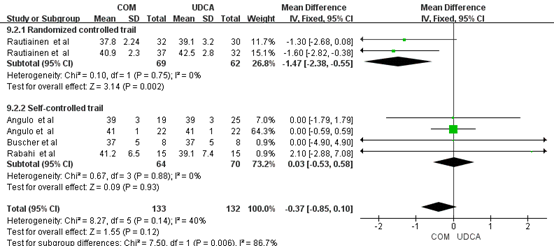


Abbreviations: UDCA, ursodeoxycholic acid; COM, combination therapy; SD, standard deviation; IV, inverse-variance; CI, confidence interval; df, degree of freedom; PBC, primary biliary cholangitis; ALB, Albumin.

**IgG and IgM**

Two trials including 84 patients reported data regarding the endpoints of IgG levels10, 13, showing that combination therapy could reduce the IgG levels of PBC patients (MD=-1.60，95% CI=-2.63，-0.57, P=0.002, Figure S4), with moderate statistical heterogeneity between the two groups (I2=44%, P=0.18).

Three trials including 92 patients reported data regarding the endpoints of IgM levels9, 10, 13, showing that combination could significantly reduce the IgM levels of PBC patients (MD=-1.28，95% CI=-2.18，-0.38, P=0.005, Figure S4), with moderate statistical heterogeneity between the two groups (I2=42%, P=0.18).

Figure S4: IgM and IgG levels of patients treated with monotherapy versus combination therapy for PBC.


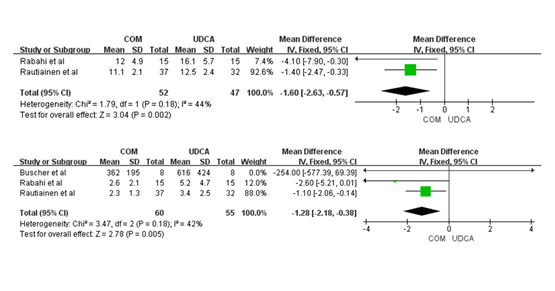


Abbreviations: UDCA, ursodeoxycholic acid; COM, combination therapy; SD, standard deviation; IV, inverse-variance; CI, confidence interval; df, degree of freedom; PBC, primary biliary cholangitis; IgM: immunoglobulin M; IgG: immunoglobulin G.
